# Poly(caprolactone)-*b*-poly(ethylene glycol)-Based Polymeric Micelles as Drug Carriers for Efficient Breast Cancer Therapy: A Systematic Review

**DOI:** 10.3390/polym14224847

**Published:** 2022-11-10

**Authors:** Siti Hajar Ahmad Shariff, Wan Khartini Wan Abdul Khodir, Shafida Abd Hamid, Muhammad Salahuddin Haris, Mohamad Wafiuddin Ismail

**Affiliations:** 1 Department of Chemistry, Kulliyyah of Science, International Islamic University Malaysia, Kuantan 25200, Pahang Darul Makmur, Malaysia; 2 Department of Pharmaceutical Technology, Kulliyyah of Pharmacy, International Islamic University Malaysia, Kuantan 25200, Pahang Darul Makmur, Malaysia

**Keywords:** PCL-PEG, polymer micelle, drug cargo, drug delivery, breast cancer

## Abstract

Recently, drug delivery systems based on nanoparticles for cancer treatment have become the centre of attention for researchers to design and fabricate drug carriers for anti-cancer drugs due to the lack of tumour-targeting activity in conventional pharmaceuticals. Poly(caprolactone)-*b*-poly(ethylene glycol) (PCL-PEG)-based micelles have attracted significant attention as a potential drug carrier intended for human use. Since their first discovery, the Food and Drug Administration (FDA)-approved polymers have been studied extensively for various biomedical applications, specifically cancer therapy. The application of PCL-PEG micelles in different cancer therapies has been recorded in countless research studies for their efficacy as drug cargos. However, systematic studies on the effectiveness of PCL-PEG micelles of specific cancers for pharmaceutical applications are still lacking. As breast cancer is reported as the most prevalent cancer worldwide, we aim to systematically review all available literature that has published research findings on the PCL-PEG-based micelles as drug cargo for therapy. We further discussed the preparation method and the anti-tumour efficacy of the micelles. Using a prearranged search string, Scopus and Science Direct were selected as the databases for the systematic searching strategy. Only eight of the 314 articles met the inclusion requirements and were used for data synthesis. From the review, all studies reported the efficiency of PCL-PEG-based micelles, which act as drug cargo for breast cancer therapy.

## 1. Introduction

The statistics from the International Agency for Research on Cancer in 2020 have estimated around 19.3 million new cases and almost 10.0 million deaths worldwide, with female breast cancer as the most commonly diagnosed cancer [1]. The World Health Organization in 2019 reported cancer as the first or second most significant cause of mortality among people before the age of 70 in 112 of 183 countries [2]. According to the National Cancer Institute, some cancer treatments include surgery, chemotherapy, immunotherapy, stem cell transplant, and precision medicine [3]. Among the many treatments, chemotherapy is one of the most commonly used methods for cancer therapy [4].

Nevertheless, several drawbacks have limited the benefits of chemotherapy treatment, such as the poor water solubility of some anti-cancer drugs that lower their efficiency. In addition, anti-cancer drugs also possess a high toxicity that results in severe side effects which cannot be reduced using traditional pharmaceutical dosage forms [5]. While conventional pharmaceutical formulations are lacking in the activity that targets tumours, resulting in only a limited amount of drugs that enter systemic circulation to target the tumour tissues. Consequently, the drug uptake of conventional pharmaceuticals is lowered [6]. As such, drug delivery systems based on nanoparticles for cancer treatment have become the centre of attention for researchers to design and fabricate drug carriers for anti-cancer drugs, such as vesicles, liposomes, nanogels, and polymer micelles [5,7,8]. These nanodrug delivery methods can lower drug toxicity while increasing bioavailability since the drugs are dissolved, adsorbed, and covalently bound to the surface of the nanocarriers. The surface of the carriers can also be modified to direct the drug toward the tumour, minimising drug transport to healthy tissues and increasing treatment safety. The discovery of this cancer-targeting technology based on nanodrug delivery has resurrected the medicinal use of many powerful anti-cancer drugs that previously contained a high toxicity [6,7,9].

Researchers highly sought polymer micelles in breast cancer treatment due to their flexibility in designing and modifying their structures and compositions [10]. As a result, the development of polymer micelles as breast anti-cancer drug carriers has been ongoing even up to this day. Polymer micelles are usually spherical, nano-sized, amphiphilic copolymers that self-assembled spontaneously in aqueous media to form micelles above the critical micelle concentration (CMC), comprised of hydrophobic and hydrophilic block domains. The hydrophobic core dissolves the hydrophobic drugs, improving their solubility and biostability. At the same time, the hydrophilic outer shell provides micelles with compatibility in the aqueous media and shields the drugs in the core from interactions with the blood components [11].

One of the most commonly used polymeric micelles is based on a polyester-polyether, poly(caprolactone)-*b*-poly(ethylene glycol) (PCL-PEG) block copolymer due to their amphiphilicity, high biocompatibility, controlled biodegradability, and self-assembling ability to produce polymeric micelles in aqueous media [12,13,14,15]. Polyester cores were reported to have higher hydrophobic anti-cancer drug loading than polyether [16]. PCL has a very low glass transition temperature, making it suitable for developing drug delivery systems based on nanoparticles, in addition to its biodegradability, biocompatibility, and FDA approval [17]. Compared to other hydrophobic polyesters such as poly(lactic-co-glycolic acid (PLGA) and polylactic acid (PLA), PCL displayed a relatively weak acidic environment, lessening the biological inflammation. Furthermore, PCL is less expensive and more stable in the body than PLGA and degrades rather slowly [18]. PCL is the most promising polyester for the development of novel, commercial medical devices. This capability is related to PCL’s unique physicochemical features, relatively harmless biodegradation behaviour, and the ability to fine-tune and make significant chemical alterations [19].

On its own, pure PCL has poor water stability as it easily aggregates. Therefore, the addition of PEG chains is used to address this issue. PEG has been used as a therapeutic agent for a long time as an FDA-approved hydrophilic constituent of polymeric micelles because it can prevent micelle uptake in the reticuloendothelial system (RES), hence lengthening the blood circulation time of the drug in the polymer micelles [20,21]. PEGylation of the hydrophobic PCL results in amphiphilicity properties and a controllable degradation speed and drug release profile, enhancing its biocompatibility and circulation time [17]. Apart from that, the addition of PEG to PCL increased the degree of crystallinity of PCL, resulting in more refined PCL crystals. In fact, even a substantial amount of PEG resulted in a significant decrease in the degradation temperature, crystallinity, time of crystallisation, and an increase in the crystal’s average size [22]. The synergistic effects of PCL-PEG copolymers make them attractive in the anti-cancer drug delivery system. Over the years, the application of PCL-PEG-based polymeric micelles in the area of cancer therapeutics has been studied numerous times as drug carriers for various types of cancer, such as breast cancer [23,24], colorectal cancer [25], lung cancer [26], colon cancer [27,28], prostate cancer [29], and others. PCL-PEG based nanoparticles as nanocarriers for chemotherapeutic drugs such as paclitaxel, campothecin, and doxorubicin confirmed increased cellular internalisation, sustained drug release, and lower cytotoxic effects compared to free drugs [30].

These studies focus on the efficacies and capabilities of the amphiphilic polymeric micelles to act as anti-cancer drug cargo. However, there is still a limited number of existing studies that have been reviewed systematically, specifically on breast cancer therapy. Hence, this review was conducted to systematically review past studies in detail. The fundamental question that led to this review is: what is the efficiency of PCL-PEG-based micelles as drug carriers for breast cancer therapy? This study aims to narrow the gap by systematically examining past related studies (2016–2021) to acquire better knowledge on the efficiency of the PCL-PEG-based micelles as drug carriers for breast cancer treatment.

## 2. Methods

In this section, the reviewers discussed the strategy used to identify papers about poly(caprolactone)-*b*-poly(ethylene glycol)-based polymeric micelles as drug carriers for efficient breast cancer therapy. The method used for this systemic review was PRISMA, which included Scopus and ScienceDirect as resources.

### 2.1. The Review Protocol—PRISMA

This systematic review uses the Preferred Reporting Items for Systematic Reviews and Meta-Analyses Protocols (PRISMA) guideline. PRISMA is a published standard that guides researchers on how to conduct a systematic literature review. It is widely used in medical research and can identify the inclusion and exclusion criteria of the study [31].

### 2.2. Formulation of the Research Question

The research question for this review was formulated based on PICO. PICO is a method that helps construct a relevant research question for systematic literature reviews. The four main concepts in PICO are Population or Problem, Intervention or Experimental Variables, Control Variable, and Outcome [32]. Based on the concepts, the researchers outlined four main aspects in the review: PCL-PEG (population), micelle (Intervention/experimental variables), drug carrier (control variable), and breast cancer (outcome), which then guided the formulation of the leading research question: what is the efficiency of the PCL-PEG-based micelles as drug carriers for breast cancer therapy?

### 2.3. Resources

Two electronic databases were used as sources for this study: Scopus and ScienceDirect. These databases are relevant and provide high-impact factor publication [33]. The researchers analysed the titles and abstracts of the published articles according to the inclusion criteria in this study.

### 2.4. Systematic Searching Strategies

The systematic search process for selecting relevant articles for this review was done in three stages: identification, screening, and eligibility.

#### 2.4.1. Identification

In the first stage, the keywords were identified and expanded by looking for similar or relevant terms in dictionaries, thesauruses, and previous research. To make the search process more accessible and to limit the results to relevant articles, a combination of symbols and coding, such as field codes, Boolean operators (AND, OR), wildcards, and truncation, were used to connect the keywords. The search strings were developed and used on Scopus and ScienceDirect after all keywords were determined (Table 1). Different search strings were used between Scopus and ScienceDirect due to some of the characters not being accepted as keywords.

#### 2.4.2. Screening

A total of 314 articles were automatically screened using the sorting function available in the databases by selecting the predefined inclusion and exclusion criteria (Table 2). The first criterion decided was the article category, with the researchers agreeing to focus solely on research articles because they are classified as primary sources and provide actual data [31,34]. As a result, publications other than research articles were excluded from the current evaluation, including systematic reviews, review papers, meta-analyses, meta-syntheses, proceedings, books, book chapters, and book series. Aside from that, the current study solely looked at articles written in English. Therefore, publications in other languages were not considered. Moreover, articles’ acceptable timeline to be included in the review was six years (2016–2021). There were 240 publications removed from the study because they did not fit the criteria. Five articles were identified to be duplicated during the screening process and thus were removed. The remaining 69 articles were found and prepared for the next step in the process: eligibility.

#### 2.4.3. Eligibility

The researchers manually examined the remaining 69 articles to ensure they were fit to be included in the present study to achieve the study’s objectives by thoroughly reading the articles’ titles and abstracts. A total of 61 articles were excluded because the articles focus on other types of cancers rather than breast cancer, other types of copolymers rather than PCL-PEG copolymers, and polymersomes and nanoparticles rather than micelles. As a result, eight articles were selected for the next process: quality appraisal.

### 2.5. Quality Appraisal

Quality appraisal was conducted to assess the quality of the articles’ content. Two authours independently examined eight papers and categorised them as high, medium, or poor quality based on the pre-set criteria. The criteria were developed in response to the systematic review’s research questions. Mutual agreement between authours was practiced during the rating process to eliminate bias. As a result, data abstraction and analysis were carried out in eight papers.

### 2.6. Data Abstraction and Analysis

The data abstraction was done in response to the formulated research question that had been developed. Any data from the reviewed researchers that could be used to answer the research question was retrieved and tabulated. Thematic analysis was then used to find the themes and sub-themes from the abstracted data based on patterns, similarities, and linkages. The creation of topics was the initial step in the thematic analysis. The researchers attempted to find the patterns that appeared to connect the abstracted data from all eight pieces of study in this step. Any related or comparable data was organised, and five themes were eventually produced. The researchers repeated the process for each new theme, yielding six sub-themes in all (Table 3). The researchers reviewed the data’s accuracy and discussed any anomalies in the resulting themes and sub-themes with one another to verify the data’s relevance and reliability. The researchers then named the five themes: synthesis and characterisation of PCL-PEG, preparation of micelles, characterisation of micelles, drug delivery study, and anti-tumour efficacy (Table 3).

## 3. Results

### 3.1. Selected Articles’ Background

**Figure 1:**
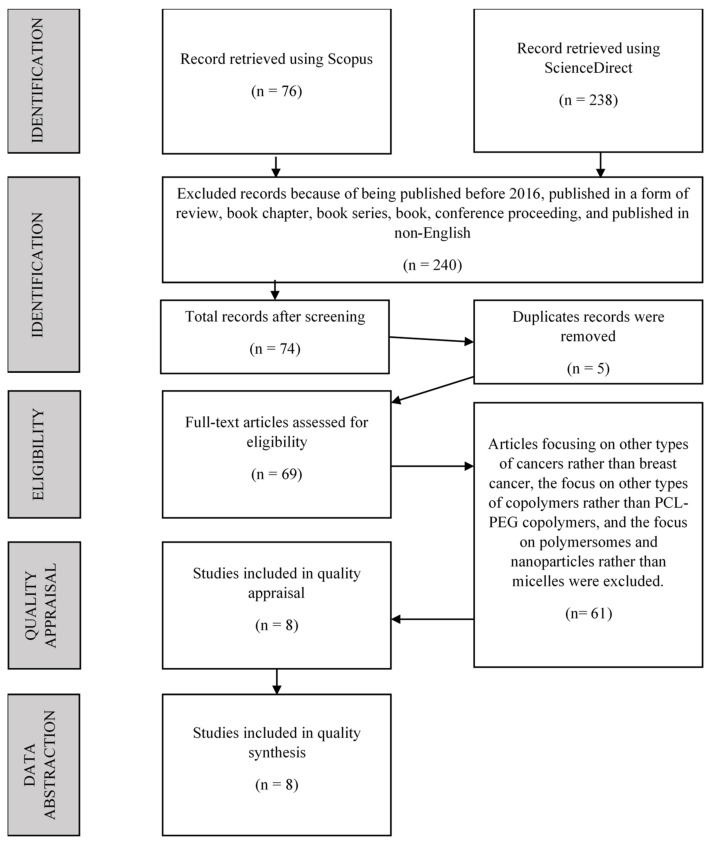
The flow diagram of the systematic literature review (adapted from Mohamed Shaffril et al. [31]).

Eight selected articles were successfully obtained for the current review (Figure 1). Five themes were developed based on the thematic analysis: synthesis and characterisation of PCL-PEG, preparation of micelles, characterisation of micelles, drug release study, and anti-tumour efficacy. Six sub-themes resulted from further study of the themes. From the eight selected papers, one was published in 2016 [35], four were published in 2017 [36,37,38,39], two were published in 2018 [40,41], and one was published in 2019 [42].

### 3.2. Themes and Sub-Themes

#### 3.2.1. Synthesis and Characterisation of PCL-PEG Copolymers

**Figure 2:**
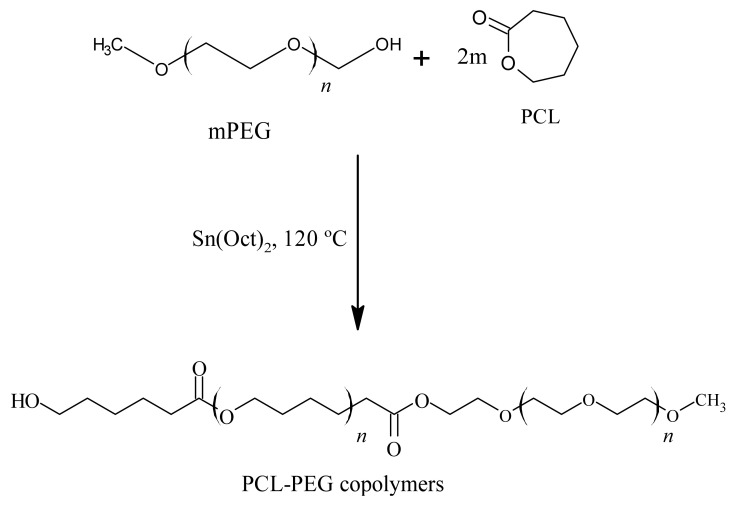
Schematic route for the preparation of PCL-PEG copolymers.

**Figure 3:**
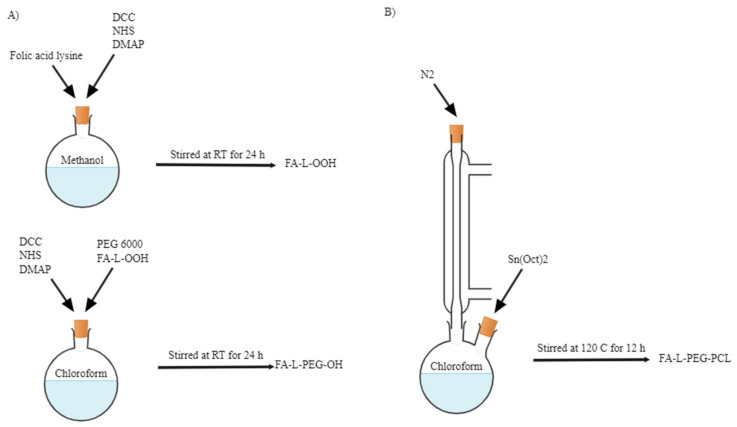
Preparation of (**A**) FA-L-PEG-OH and (**B**) FA-L-PEG-PCL. Reprinted/adapted with permission from Elsevier [40].

Seven studies synthesised the PCL-PEG copolymers, except for Peng et al., which used pre-synthesized PCL-PEG copolymers. The copolymers were synthesised by using ring-opening polymerisation (ROP) of ε-caprolactone (ε-CL) [35,36,37,38,39,40,41]. In most studies, the PEG with the hydroxyl end group was used to initiate the ring opening of ε-CL in the presence of the catalyst stannous octoate (Sn(Oct)_2_) and under a nitrogen atmosphere (Figure 2) [35,36,37,39,40,41]. However, Mahdaviani et al., initiated the ROP of ε-CL by utilising N-protected 3-aminopropan-1-ol, and PCL-PEG copolymer was synthesised via an amidation reaction between the -COOH groups of PCL and the -NH3 end of PEG [38]. In addition, Zamani et al., added folic acid (FA) as a molecular probe to the synthesised PCL-PEG copolymers for targeted cancer treatment in the form of folate-lysine-PCL-PEG (FA-L-PEG-PCL) (Figure 3). To ensure sufficient conversion of ε-CL to PCL, the reaction was heated at 110 °C for 24 h [38]. However, most studies conducted the reaction at 120 °C to shorten the reaction period to 12 h. Various molecular weight copolymers were produced by manipulating the ε-CL: PEG ratio. The summary of the reaction condition is tabulated in Table 4.

Each study employed Fourier transform infrared spectroscopy (FTIR), proton nuclear magnetic resonance spectroscopy (1H NMR), and gel permeation chromatography (GPC) to characterise the synthesised copolymers. The successful formation of PCL-PEG copolymers in the studies was indicated by the sharp and intense bands around 1720.0 cm^−1^ and 1100.0 cm^−1^ of the FTIR analysis, representing the carboxylic ester (C=O) and ether (C–O) groups of the PCL-PEG linkage. The NMR analysis from the studies revealed that the peaks around 2.2–2.5 ppm of methylene proton –OCCH_2_- and 4.06 ppm of methylene proton –CH_2_OOC– belongs to the linkage between the PCL and PEG, which confirmed the formation of PCL-PEG copolymers. Mahdaviani et al., found that the synthesised copolymers had a 1:1 ratio of PCL and PEG by assessing the peak area associated with the PEG methylene group to that of PCL [38]. The ratio of the copolymers can be determined by equating the peak integration area at ~4.06 ppm (PCL block) to that at ~3.6 ppm (PEG block) [39]. Meanwhile, the number average molecular weight (M_n_) of the PCL-PEG copolymers can be determined based on the equation:

M_n_ = (1 + R) × PEG

where R = the ratio of the integration area of PCL:PEG and PEG = the number average molecular weight of PEG.

GPC analysis from all studies showed a polydispersity index (PDI) closer to one (<1.4), indicating the uniform distribution of the polymerisation of PCL-PEG copolymers. Hu et al., also stated that the study’s unimodal curve of the GPC chromatogram suggested no impurities in the polymers [39].

**Figure 4:**
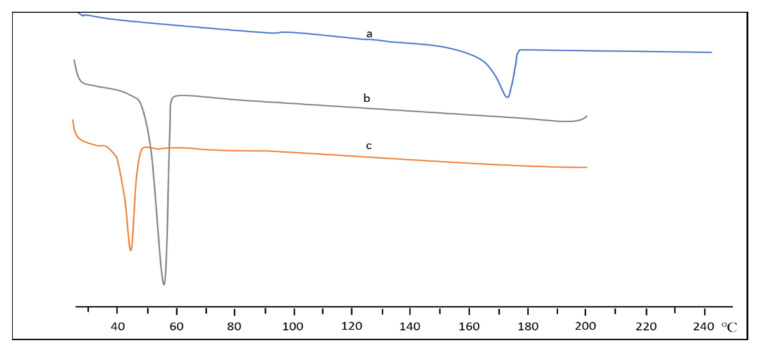
DSC thermogram of (a) CUR (b) mPEG-PCL, and (c) CUR/mPEG-PCL micelles. Reprinted/adapted with permission from Elsevier [35].

Thermal analysis of the synthesised PCL-PEG was done using differential scanning calorimetry (DSC) analysis. The DSC thermograms in each study recorded an endothermic peak for the melting point of the copolymers. The melting transition temperatures of PCL and PEG homopolymers that were greater than those of respective blocks in copolymers indicated the interaction between the PCL and PEG blocks [39]. In addition, the formation of two peaks during the cooling process in the thermogram was most likely due to the length of the PCL block, which was half of the PEG block. The PEG block’s higher crystallisation temperature compared to the PCL block resulted in the appearance of two peaks in the thermogram. However, when the PEG block’s molecular weight and the PCL block’s molecular weight were close enough, their peaks merged and became exothermic peaks. The fact that the crystallisation temperature of PEG was more significant when the block length was longer supports the conclusion that crystallisation temperature is positively related to the block length [39]. The same pattern was observed by Kheiri Manjili et al. [35,36,37,41] where the DSC thermogram copolymers showed two merged endothermic peaks of PCL and PEG (Figure 4). The characterisation of PCL-PEG copolymers is summarised in Table 4.

#### 3.2.2. Preparation of Micelles

The selected studies adopted four types of micelle preparation methods: nanoprecipitation, thin-film hydration, co-solvent evaporation, and emulsion solvent evaporation. The preparation methods of the micelles are summarised in Table 5. The suitable techniques were chosen based on various variables such as the particle’s size, the active agent’s stability, and the finished product’s toxic effect [38]. In addition, PCL-PEG copolymers can self-assemble in appropriate solvent conditions because of their duality and are able to transform into different types of structures, such as spherical, cylindrical, and others, depending on the optimisation of the solvent conditions [41]. Solvents such as acetone, methylene dichloride, and chloroform were used to dissolve both PCL-PEG copolymers and hydrophobic drug models before adding water as the aqueous phase for the copolymers to self-assemble into micelles. Meanwhile, Mahdaviani et al., optimised the polymeric micelles’ preparation method regarding the carrier size and encapsulation efficacy by modifying the organic: aqueous phase ratio and the sequence in which the phases were added. They found that the optimised ratio was 10% (*w*/*w*) of the organic to aqueous phase [38]. In contrast, Peng et al., optimised the PCL-PEG chain length to encapsulate the hydrophobic drug into the semi-crystalline PCL core and to promote self-assembly into specific morphology. The weight ratio of 3:7 of PCL-PEG copolymers was reported as the optimised matrix to encapsulate the drug and self-assemble it into worm-like micelles. The ratio was chosen as the ideal formulation due to the micelles’ smaller and homogeneous size distribution [42].

Two studies adopted functionalised conjugations to the micelles for the targeted delivery of drugs. Mahdaviani et al., used a peptide-functionalized ligand, a cyclic ten amino acid peptide (GCGNVVRQGC), which was a tumour metastasis-targeting (TMT) peptide to promote the effective targeting of the anti-tumour CBZ. The TMT peptide was conjugated onto PCL-PEG micelles via the carboxyl group PCL-PEG copolymer’s covalent bonds. The Moc-protected TMT peptide was linked to PCL-PEG using EDC and NHS [38]. In another study, Peng et al., used Herceptin (HER) as antibody-conjugated nanoparticles (ACN) to bind extracellularly to the p185 glycoprotein domain of the HER2-positive breast cancer receptor to cause apoptosis in tumour cells or to stop the cell cycle progression. The drug-loaded PCL-PEG-HER was prepared by performing a Schiff base reaction between the aldehyde group of CHO-PCL-PEG anchored at the surface of the drug-loaded micelles with the amine group of HER using a molar ratio of 5:1 aldehyde to amine. Then, the -C=N- was reduced to -C-N- by using CH3BNNa [42].

#### 3.2.3. Characterisation of Micelles

The determination of the micelles’ morphology was common under this theme, followed by the distribution of particle size and zeta potential, drug loading and encapsulation efficiency, and thermal analysis. Table 5 summarised the characterisation of the selected micelles.

##### Morphology of Micelles

**Figure 5:**
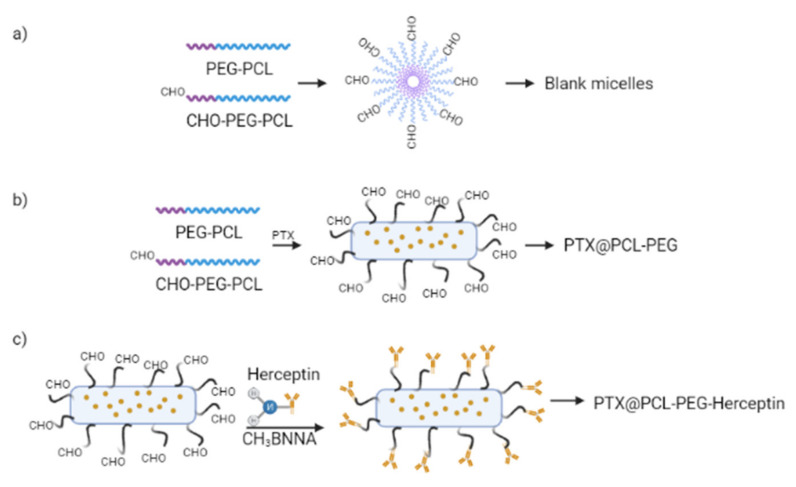
The formulation strategy of (**a**) Blank micelles, (**b**) PTX/CL-PEG, and (**c**) PTX/PCL-PEG-Herceptin. Reprinted/adapted with permission from Elsevier [42].

In each study, the morphological characterisation of the micelles was examined by utilising atomic force microscopy (AFM) [35,36,37,40,41], transmission electron microscopy (TEM) [39,42], and scanning electron microscopy (SEM) [38]. The PCL-PEG micelles demonstrated a uniform spherical shape when observed using atomic force microscopy (AFM). TEM results revealed that most nanoparticles had a distinct spherical shape and uniform size [39]. Hu et al., reported that when the PCL-PEG block ratio was 1, the copolymers formed polymeric micelles. Meanwhile, Peng et al., found the self-assembled spherical amphiphilic PCL-PEG micelles became worm-like micelles when PTX was loaded into the PCL-PEG micelles in an aqueous solution (Figure 5). After conjugation with a particular number of HER molecules, the worm-like structure of the micelles was preserved [42]. Meanwhile, the SEM image in the study showed that CBZ polymeric micelles had a uniform spherical morphology, with a particle size of around 100 nm and a small particle size range [38].

##### Particle Size and Zeta Potential

In each study, the size of the micelle observed by AFM, TEM, and SEM was somewhat smaller compared to DLS because the diameter calculated by DLS represents the hydrodynamics diameter. In contrast, the diameter acquired by AFM, SEM, and TEM was measured after the evaporation of water. Kheiri Manjili et al., found that the CUR/PCL-PEG micelles’ stability was reduced when the particle size and the polydispersity index (PDI) were increased [35]. In another study, the micelles were unstable in water when the ART/PCL-PEG mass ratio was 1 due to ART’s hydrophobicity, guiding more drugs at high concentrations to be adsorbed onto the micelle’s exterior [37]. Hu et al., found that increasing the length of the hydrophobic PCL block increased the size of the particles for two types of nanoparticles with a fixed PEG block [39], while for a sequence of nanoparticles with fixed PCL:PEG ratios, the diameters of nanoparticles increased when the molecular weight of the copolymers increased. Meanwhile, Mahdaviani et al., found that the particle size of the copolymers had a unimodal distribution, which was advantageous in terms of providing a more extended pharmacological profile in vivo [38]. Furthermore, Peng et al., reported that the PTX wrapping significantly increased the micelles’ average diameter, and HER conjugation onto the surfaces expanded the micelles’ size marginally [42]. Additionally, all studies reported a slightly negative zeta potential of the copolymeric micelles. Kheiri Manjili et al., discovered that a minor negative charge surface of the SF/PCL–PEG–PCL micelles can increase the drug’s circulation time [36]. Similarly, Peng et al., discovered that the slightly negatively charged PTX/PCL-PEG-HER was more ideal for blood persistence and nano complexes contacting the cell surface than their negatively charged counterparts [42].

##### Drug Loading (DL) and Encapsulation Efficiency (EE)

The two methods used to measure the DL and EE were high-performance liquid chromatography (HPLC) and UV-Vis spectrophotometry. The various parameters for the HPLC analysis are summarised in Table 6. The wavelength of 420 nm was used for CUR. In contrast, the wavelengths of 284 nm and 244 nm were used for atorvastatin and rosuvastatin, respectively, and the wavelength of 278 nm was used for TMX in the UV-Vis analysis [35,40,41].

The DL and EE were calculated using the following equations:

$$\mathrm{DL}\;\left(\%\right)=\frac{\mathrm{weight}\;\mathrm{of}\;\mathrm{drug}\;\mathrm{in}\;\mathrm{micelles}}{\mathrm{weight}\;\mathrm{of}\;\mathrm{micelles}}\times 100$$

$$\mathrm{EE}\;\left(\%\right)=\frac{\mathrm{weight}\;\mathrm{of}\;\mathrm{drug}\;\mathrm{in}\;\mathrm{micelles}}{\mathrm{weight}\;\mathrm{of}\;\mathrm{initial}\;\mathrm{drug}}\times 100$$

Different mass feed ratios of drug/PCL-PEG were tested to improve the development parameters and investigate the influence of the drug/copolymer ratio on the drug loading and encapsulation efficiency of CUR, SF, and ART [35,36,37]. Kheiri Manjili et al. [35,36,37] found that different drug/PCL-PEG ratios were found to give other micelles stability. For example, increasing the particle size and polydispersity index (PDI) reduced the strength of the drug/PCL-PEG micelles. Besides, the mass ratio of 0.75 and 1 of drug/PCL-PEG resulted in instability of the micelles in water due to aggregation of the micelles. Thus, the drug/PCL-PEG mass ratio at 0.25 was preferred.

Additionally, the effects of the CL:PEG ratio on the DL and EE were also investigated to develop an ideally high DL or EE. The DL and EE of micelles with fixed PEG length were found to increase when the PCL length increased [35,36,37,39]. This observation was attributed to the encapsulation of the hydrophobic drug into the centre via the hydrophobic interaction of PCL and the drugs and the hydrophilic interaction between water and PEG. The DL and EE of the hydrophobic drug in PCL-PEG copolymers increased due to the strong hydrophobic interaction between the longer PCL chain and the drug. This outcome was anticipated since the encapsulation of the drugs into the hydrophobic centre increases the micelles’ volume.

Kheiri Manjili et al. [41] revealed that the drug loading of atorvastatin was greater than rosuvastatin because of its hydrophobic nature, which is preferred by the micelles. Hence, the rosuvastatin-loaded micelles appeared smaller than atorvastatin due to their increased size during drug loading. Whereas Mahdaviani et al., discovered that by encapsulating the CBZ into the micelle, the solubility of the drug in an aqueous solution was increased hundreds-fold, which, in turn, increased the drug substance at the site of action due to a high encapsulation efficiency [38].

##### Thermal Analysis

**Figure 6:**
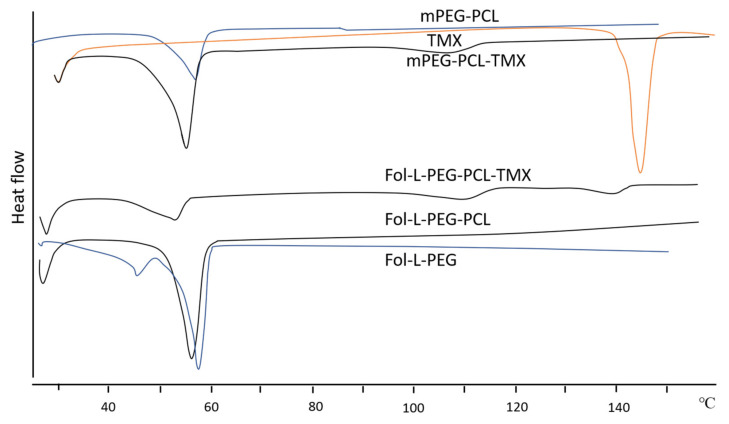
DSC curves of copolymers and drug-loaded copolymers. Reprinted/adapted with permission from Elsevier [40].

Thermal analysis of the copolymeric micelles was done using differential scanning calorimetry (DSC). In all studies, the DSC thermogram demonstrated endothermic peaks for the PCL-PEG copolymeric micelles. Zamani et al., suggested that the single peak shown on the DSC curve indicates the absence of impurities in the copolymers. A sharp endothermic peak at 142 °C (Figure 6) belongs to the melting point of free TMX, which indicates its crystallinity. After the drug and copolymers are combined, the peak vanishes as the crystalline phase changes to amorphous, indicating successful drug incorporation in the copolymers [40]. Correspondingly, Kheiri Manjili et al. [35,37,41] reported a higher thermogram display of the melting crystalline PCL block of the copolymers’ peak compared to the melting temperature of their micelles, confirming the physical interaction between the drug and PCL-PEG copolymers.

#### 3.2.4. Drug Release Study

The dialysis method was adopted in the drug release study [35,36,37,40,41,42] and no considerable initial burst of drug release was seen from the micelles. The prolonged release of the hydrophobic drugs was due to the incorporation of the drugs in the core of the micelles. The summary of the release study was tabulated in Table 7.

**Figure 7:**
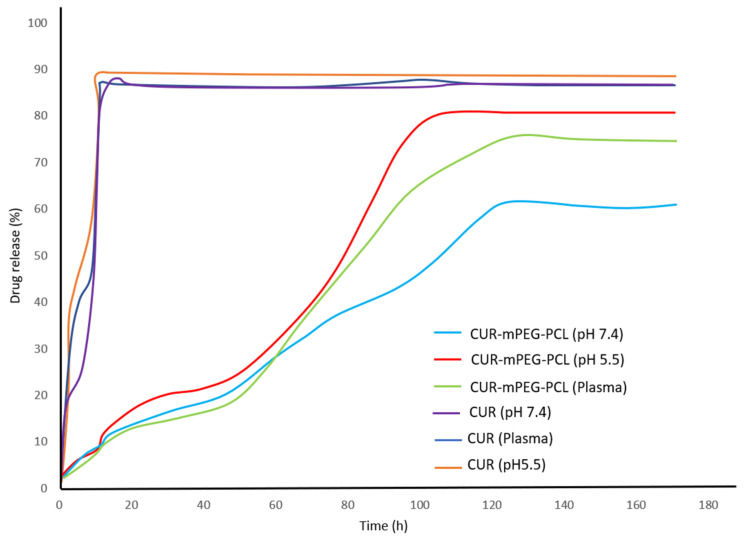
The release profile of CUR from CUR/MPEG-PCL micelles in different release media. Reprinted/adapted with permission from Elsevier [35].

A pH-sensitive release was observed in each study. Kheiri Manjili et al. [35,36,37,41] performed the drug release study on drug-loaded micelles at neutral pH (pH 7.4), acidified PBS solution (pH 5.5), and human plasma. Freshly prepared human plasma was collected from a volunteer to investigate the influence of chemical and biological parameters on the hydrophobic drug release from the micelles (Figure 7). The free drug release was done as a control to ensure that drug molecules’ diffusion over the dialysis membrane was not the rate-limiting step. They discovered that the percentage of the drugs released from the micelles increased from a pH value of 7.4 to 5.5 due to the hydrolysis degradation of copolymers in acidic conditions. Similar action was observed in the plasma medium when hydrolysis of the esoteric link of the copolymer occurred due to the enhancement of certain enzymes present in human plasma.

Comparatively, Peng et al., found that the cumulative release rate of PTX in TAXOL^®^ was higher than that of PTX/PCL-PEG and PTX/PCL-PEG-HER at pH 7.4 but showed a similar release rate for all at pH 6.5. This proved that PTX/PCL-PEG and PTX/PCL-PEG-HER had a higher stability than PTX/TAXOL^®^ in physiological conditions and that Herceptin conjugation has no influence on the release rates of PTX/PCL-PEG-HER when compared to PTX/PCL-PEG [42].

In another study, Zamani et al., found no initial burst release observed for the first three hours for the TMX/PCL-PEG micelles at pH 2. Since food and drugs pass the gastrointestinal tract in less than 2 h, this drug-loaded copolymer could be an excellent nanodrug carrier for oral application [40].

##### Bioavailability of Drugs

The bioavailability of the drugs was assessed using a pharmacokinetic study via oral [31,32] and intravenous routes [37,42] and this was analysed using HPLC and LC-MS. The pharmacokinetic study of the drugs is summarized in Table 8.

Kheiri Manjili et al., reported an increase in the bioavailability of drugs loaded in micelles compared to the free drug solution. The micelles were effective in increasing drug absorption and delaying the drug release, indicating sustained release of the drugs [35,36,37]. Meanwhile, Peng et al., found that worm-like micelles enter into the peripheral tissues more quickly than the spherical micelles based on the apparent volume of distribution (V_d_). This showed that PTX-PCL-PEG-Her possessed an improved targeting capability compared to PTX-PCL-PEG [42].

#### 3.2.5. Anti-Tumour Activity

##### Cell Viability and Cytotoxicity Study

The cancerous cell viability and cytotoxicity after treatment using drug-loaded PCL-PEG micelles were done via MTT assay [35,36,37,38,40,41,42]. The cell toxicity of the drug/PCL-PEG micelles was reported to be directly proportional to the micelles’ concentration. There was no significant difference in the anti-cancer effect of the CUR-loaded and ART-loaded copolymer micelles at different treatment times in all of the cancer lines tested (mice breast adenocarcinoma, 4T1 and human breast adenocarcinoma, MCF-7), indicating the remarkable anti-cancer effect of the drug-loaded PCL-PEG micelles for breast cancer lines [35,37]. No toxicity was recorded for the drug and blank micelles at different concentrations. However, Kheiri Manjili et al. [37] observed that the MCF-7 cell line was more susceptible to ART treatment compared to the 4T1 cell line. Meanwhile, MTT data statistics showed that, except in MCF10A cells, SF/PCL-PEG micelles significantly (*p* < 0.05) decreased cell viability at each concentration when compared to their copolymers in MCF-7 and 4T1 cancerous cells [36]. The MTT assay also showed that SF-loaded micelles enhanced the cytotoxic effect of SF in MCF-7 and 4T1 cell lines. The blank micelles’ biocompatibility as nanocarriers was evaluated by performing the MTT assay against HFF-2 (normal cell line) and MCF-7. At 48 and 72 h, minimal cytotoxic effects were seen on MCF-7 and HFF2 cell lines at the highest tested concentrations of blank polymer. All statin-loaded micelles on normal cells (HFF2) had a minimal cytotoxic effect compared to free statins.

**Figure 8:**
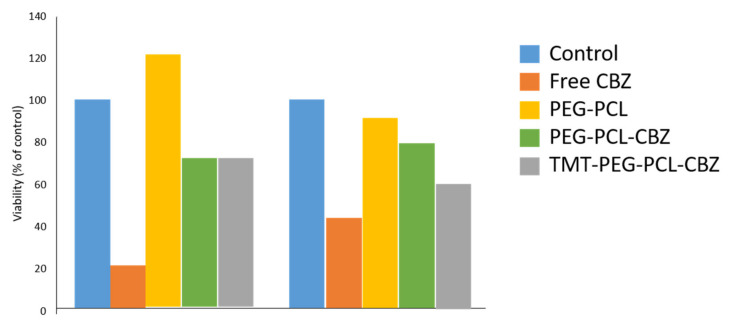
In vitro cell cytotoxicity of free CBZ and CBZ-loaded block copolymer micelles against MCF-7 and MDA-MB 231 after a 72 h incubation. Reprinted/adapted with permission from Elsevier [38].

A study by Mahdaviani et al., showed that the CBZ encapsulated in TMT-PCL-PEG showed a greater efficacy in killing highly metastatic cancer cells (MDA-MB-231) than the one encapsulated in PCL-PEG micelles alone and recorded no substantial increase in the cytotoxic effect compared to non-targeted micelles in MCF-7 cells. There was also no significant distinction in the percentage of cell viability between the blank micelles and the control (Figure 8) [38]. The same pattern was reported by Zamani et al., where TMX-loaded FA-L-PCL-PEG micelles decreased the cell viability of MCF-7 cell lines up to 53% compared to the PCL-PEG micelles alone [40], and Peng et al., where PTX-loaded PCL-PEG-Herceptin showed the highest anti-cancer effects compared to PCL-PEG and TAXOL^®^ [42]. Peng et al., also found that the typical antibody dose for in vivo anti-tumour experiments was 10 mg/kg, while Herceptin used in each formulation studied was less than 1.3 mg/kg. The results showed that Herceptin had neither cytotoxic nor therapeutic effects at the utilised dose [42].

##### Anti-Tumour Efficacy

An in vivo experiment by Kheiri Manjili et al. [35,36,37] was done by measuring the tumour growth profile of 4T1 mice breast cancer as a function of time. The mean tumour volumes of the mice treated with a low concentration of CUR/PCL-PEG micelles after 25 days (1075 ± 195 mm^3^) was comparable to the one treated with higher concentrations of free CUR (960 ± 158 mm^3^), which corresponded to 45.7% and 40% tumour volume reductions, respectively. Whereas the mean tumour volumes of ART/micelle-treated mice reached below 27 mm^3^ compared to the free ART, which was about 500 mm^3^ after 20 days, and the tumour volume of the mice treated with SF-loaded micelle decreased up to 78.5% compared to the free SF, which decreased to 49.5% only. These results showed that the drug-loaded micelle had a higher anti-tumour efficacy and more therapeutic effects than the free drug. Additionally, weight variations in all mouse groups were also measured to observe the side effects of the drugs. Mice who were given a high intravenous dose of the free drug lost significantly more weight than the control, saline-treated mice. The significant weight loss observed following high-dose treatment demonstrates the severe systemic toxicity of the free drug. In contrast, mice treated with drug/PCL-PEG micelles showed no signs of severe toxicity, although they had a similar in vivo anti-tumour efficacy to high-dose free drugs. The endurance of animals treated with drug/PCL-PEG micelles or high-dose free drugs was also found to increase significantly.

The anti-tumour efficacy of PTX/PCL-PEG-HER was studied by Peng et al., using the SKBR-3 xenograft model via in vivo experiment. The tumour growth curve revealed that PTX/PCL-PEG-HER was 2-fold and 3-fold more efficient in suppressing tumour development than PTX/TAXOL^®^ and PTX/PCL-PEG within the respective days, and 3-fold, 2.4-fold, and 3.4-fold more effective at stopping tumour development than saline, Herceptin, and blank micelles, respectively, over 48 days. The median survival time of the mice in the TAXOL^®^ group was 36 days. However, all mice in the PTX/PCL-PEG-HER group were still thriving after those days, suggesting the capabilities of PTX/PCL-PEG-HER for the suppression of tumour growth and increases in the longevity of the cancerous mice [42].

Meanwhile, an in vitro experiment was done by Kheiri Manjili et al. [41] to investigate the anti-tumour efficacy of rosuvastatin and atorvastatin and statin-loaded PCL-PEG-PCL micelles on MCF-7 cells. Typically, the presence of statins in nanoparticle form decreased MCF-7 cancer cell viability. Compared to free statins, statin-loaded micelles at the lowest tested doses were observed to generate more cytostatic effect in the MCF-7 cell line. The anti-tumour effect of atorvastatin-loaded micelles was slightly lower compared to rosuvastatin-loaded micelles. The anti-tumour effect of rosuvastatin-loaded micelles was significantly greater than the atorvastatin when observed using IC50 at a concentration of 28.5 μM (48 h) and 17.5 μM (72 h) on the MCF-7 cell line. In comparison, free rosuvastatin suppressed MCF-7 cell growth at a concentration of 30.2 µM (48 h) and 20.7 μM (72 h), respectively. These showed that lower concentrations of drug-loaded micelles were needed compared to the free drug to exhibit the tumour-suppressive effect.

Mahdaviani et al., investigated the anti-cancer efficacy by analysing the apoptotic and necrotic induction effect of CBZ-loaded copolymer micelles in MCF-7 and MDA-MB-231 cell lines using flow cytometry. The study revealed significant apoptosis (16%) and necrosis (65%) in MDA-MB-231 cells, as well as mildly elevated apoptosis (2%) and necrosis (33%) in MDA-MB-231 cells treated with CBZ-loaded PCL-PEG. Compared to the control group, there was an overall increase in the percentages of early and late apoptotic MCF-7 cells, although this increase was practically the same in the targeted and non-targeted groups. However, treatment with the targeted copolymeric micelles demonstrated better apoptosis and necrosis induction than the non-targeted copolymeric micelles for the MDA-MB-231 cell line [38].

Additionally, Hu et al., researched the efficiency of the PCL-PEG polymeric micelles as drug cargo for breast cancer cells via cellular uptake experiments. The internalisation of the drug-loaded micelles into the EMT-6 breast cancer cells was examined using confocal laser scanning microscopy (CLSM). They observed that the internalised polymeric micelles (red) successfully bypassed the EMT-6 cancer cell’s nucleus (blue) in the cytoplasm after 4 h of incubation and increased in number after 24 h (refer to Figure 2 from the previous study [39]), indicating the high cellular uptake efficiency of the copolymeric micelles towards breast cancer cells [39].

## 4. Discussion

Five themes and six sub-themes were developed from the thematic analyses. Further discussion of the developed themes is presented in this section. Except for Mahdaviani et al. [38], who utilised N-protected-3-aminopropan-1-ol as the initiator for the ROP of ε-CL to PCL, the synthesis process of PCL-PEG copolymers of other studies was done using PEG as the initiator in the presence of stannous octoate as the catalyst [35,36,37,39,40,41]. The reaction had to be conducted at 110 °C to ensure complete conversion of ε-CL to PCL. The ratio of ε-CL:PEG was controlled in the studies to produce copolymers with different molecular weights.

The micelles were prepared by nanoprecipitation, thin film hydration, co-solvent evaporation, and emulsion evaporation methods. The nanoprecipitation method was chosen due to its simplicity and its ease of scaling up [35,36,37,41]. The PCL-PEG micelles produced were biocompatible, biodegradable, stable in blood, and small in size, making them an exceptional choice for drug delivery systems. The co-solvent evaporation method has the advantage of scalability and minimal drug loss during the encapsulation process compared to the dialysis method [38]. The three main aspects of optimising the size of the polymeric micelles and drug encapsulation efficiency in the co-solvent evaporation method are the type of organic co-solvent, the volume fraction of the organic: aqueous phase during the production, and the sequence to add the organic and aqueous phase. Mahdaviani et al., stated that the best organic:aqueous phase ratio for the polymeric micellar carrier to function optimally is 10% *w*/*w*. Additionally, PCL-PEG chain length can be optimised to stimulate the micelles to self-assemble into specific morphology [38]. Peng et al., reported the optimised matrix of the chain length of PCL-PEG copolymers at a weight ratio of 3:7 to encourage the hydrophobic drug to self-assemble and wrap into worm-like micelles. Interestingly, they found that the micelles’ morphology changed from spherical to worm-like structures after 6.3% of the drug was encapsulated into the micelles, which indicates that the drug can influence the morphology of the micelles [42]. Apart from that, micelles conjugated with functionalised ligands can be prepared for drug delivery to a specific target. Mahdaviani et al., found that the conjugated metastatic cancer-specific TMT ligand polymeric micelles displayed effective in vitro anti-tumour activities in highly metastatic MBA-MD-23 breast cancer cell lines but not in non-metastatic MCF-7 cell lines [38]. Correspondingly, the conjugation of Herceptin ACN to the PCL-PEG copolymers [42] modified the antibodies’ spectra from a beta-tum to a polypro II helix confirmation but still retained the targeting ability to bind to overexpressed HER2 receptors on the surface of SKBR-3 human breast cancer cell lines [42].

PCL-PEG copolymers can self-assemble into different structures depending on certain conditions. Hence, the morphology determination of the micelles was essential to observe the specific morphology the micelles assembled into. The critical parameter that affects the morphology of the self-assembled polymeric micelles is the mass or volume fraction of the PEG fraction of the PCL-PEG copolymers. Hu et al., expressed that if the PEG fraction fell between 45% and 55%, the cylindrical micelles were likely to form, while if the PEG fraction was between 55% and 70%, then spherical micelles were more likely to develop [39]. All studies exhibited spherical morphology of the polymeric micelles when observed using AFM, TEM, and SEM. A visible core-shell structure for the polymeric micelles was reported, where the PCL is the inner hydrophobic part acting as the core, encircled by the hydrophilic PEG corona as the shell of the structure. The surface of the micelles is closed since both terminals of the PEG block are attached at the core/shell interfaces, limiting the potential of opsonisation. Finally, the hydrophobic drugs are encapsulated into the PCL’s hydrophobic core through hydrophobic interactions. Additionally, Peng et al., observed worm-like micelles when the PCL-PEG micelles encapsulated the drug. As a drug cargo, the poorly water soluble PCL-PEG worm-like micelles were reported to be more efficient than spherical micelles at exiting the body’s mononuclear phagocyte system, thus loading and delivering a more significant amount of drugs to tumours and reaching farther into tumours with a higher penetration ratio, leading to possible tumour shrinkage [42].

The particle size of the micelles observed using the DLS technique showed a slightly bigger diameter than the one observed via AFM, TEM, and SEM. The surface charge of the micelles showed that the PCL-PEG micelles possess a slightly negative charge. The surface charge influences the behaviour of the oppositely charged cell membrane, whether they cluster in the blood flow, stick to, or interact with it. Because plasma and blood cells are constantly negatively charged, nanoparticles with a slightly negative surface charge might reduce nonspecific contact with them via electrostatic forces.

The capacity of drug loading and encapsulation efficiency are crucial variables for nanoparticles. A decent drug delivery system must always have a high capacity for drug loading (DL) and encapsulation efficiency (EE) [39]. Due to their hydrophobic interaction, the PCL-PEG micelles showed a high capacity for hydrophobic DL and EE. Increasing the PCL length of the PCL-PEG copolymers will improve the DL and EE of the hydrophobic drug because of the stronger hydrophobic interaction between the longer PCL chain and the drug. The incorporation of the drugs into the micelles can be observed by increasing the volume of the micelles. It is also worth noting that the PCL-PEG micelles favoured hydrophobic drugs more than hydrophilic drugs, and the encapsulation of the drugs into the PCL-PEG micelles resulted in the increase in the solubility of the drug at the site of action.

The in vitro drug release study revealed that the PCL-PEG micelles can slowly release the drug into the medium, indicating the ability of the drug-loaded PCL-PEG micelles to increase in vivo systemic circulation, i.e., the t1/2 of the drug. The prolonged drug release observed from the copolymeric micelles was because of the diffusion of the drug from the micelles and the degradation or hydrolysis of the micelles. The drug released from the PCL-PEG micelle was pH-sensitive. The drug was released faster in acidic pH than in neutral pH due to the protonation of the polymer, making the polymer matrix swell and degrade. This feature is highly desirable in numerous applications, particularly in anti-cancer drug delivery, since the microenvironments of tumour extracellular spaces, intracellular lysosomes, and endosomes are acidic; hence, the drug release from micelles may be facilitated. The resulting copolymeric micelles are highly appealing to the time-controlled administration of hydrophobic drugs for various medicinal purposes.

Cell viability and cytotoxicity studies were done to examine the in vitro anti-tumour effects of the drug-loaded PCL-PEG micelles against selected breast cancer cells. The drug-loaded PCL-PEG micelles’ increased cytotoxicity compared to the free drug can be attributed to their differential cellular absorption. This was because the internalisation of the free drug was diffusion-mediated, which was restricted after reaching saturation in the cytoplasm, resulting in minimal drug uptake compared to drugs loaded in PCL-PEG micelles. The endocytosis pathways were likely involved in the increased absorption of drug-loaded micelles and subsequent long-term drug release [35,36,37]. On the contrary, Mahdaviani et al., listed three pieces of information derived from the cytotoxicity results of the drug loaded in the conjugated polymeric micelles. First, there was no substantial change in the viability percentage between the blank micelles and the control group, indicating that the blank micelles were biocompatible. Second, the free drug interacts directly with the cells without experiencing the release process, making them more cytotoxic than the drug encapsulated in polymeric micelles. This was because the highly hydrophobic free drug can readily penetrate the lipid membranes and subsequently diffuse into the cells, resulting in a more significant cellular build-up and, consequently, increasing the cytotoxicity. Since the efficient delivery of the drug was often hindered by its hydrophobicity, using amphiphilic PCL-PEG micelles as a potent drug delivery vehicle is essential due to their biocompatible and anti-biofouling characteristics. Third, the conjugation of the cancer-specific ligand to the polymeric micelles resulted in an efficient in vitro anti-tumour effect in the specific cancer cell line [38].

Lastly, a different mode of analysis was done in each study to examine the breast anti-tumour efficiency of the drug-loaded PCL-PEG micelles. The much greater anti-tumour efficiency of drug/PCL-PEG micelles compared to the same dose of the free drug was attributed to the more extended drug circulation in the plasma and the enhanced drug accumulation in the tumour. The sensitive hydrophobic drug-loaded PCL-PEG micelles have the benefits of more extended blood circulation, prevention of RES absorption, and passive targeting of polymeric micelles to tumour tissues via the EPR effect. Also, signs of severe toxicity, such as weight loss, were absent because of the extended half-life in blood circulation and improved tumour localisation of conjugate-associated drugs [35,36,37]. Meanwhile, the flow cytometry analysis by Mahdaviani et al., showed that the active targeted group (CBZ-TMT-PCL-PEG) induced a greater percentage of apoptosis than the passive targeted group (CBZ-PCL-PEG) for the same cell line. This was due to polymeric micelles with tumour cell-targeting ligands that can facilitate drug binding and internalisation into tumour cells, which increased the drug accumulation in the tumour and improved the anti-cancer effect [38]. Moreover, Hu et al., stated that the high-efficiency cellular uptake of the PCL-PEG copolymeric micelles into the breast cancer cell line was due to the ability of the micelles to harness a nonspecific endocytosis pathway to internalise via lipid bilayer cellular membranes, depending on their shape and size [39].

## 5. Recommendation

This systematic literature review gives rise to several recommendations to guide researchers in their future studies. First, future studies should focus on the synergistic anti-cancer activity of conjugated PCL-PEG copolymers with drugs to clarify whether the conjugation functions only target specific cancerous cells in the anti-cancer treatment. Second, the effects of various morphologies, such as the worm-like structure of the micelles, should be further studied to optimise their efficacy as drug cargo. Next, more types of anti-cancer drugs and other types of cancer cell lines should be investigated to determine their anti-cancer effect and cytotoxicity on the cancer cell line since the current study only focused on a breast cancer cell line and a few drugs such as curcumin, sulforaphane, artemisinin, atorvastatin, rosuvastatin, cabazitaxel, paclitaxel, and tamoxifen.

To advance the therapy provided in the clinic, the micelles’ interactions with the complex environment and barriers that determine the fate of the micelles’ drug carrier in the body and the final therapeutic efficiency must be studied. This is because, once they enter the blood circulation, the micelles face and interact with complex biological environments such as blood component, phagocytosis, and biodistribution before arriving at the lesion site, which might modify their capability to target and transport the anti-cancer drug [43]. Additionally, micellar stability is significant in determining the delivery efficacy since micelles become unstable when facing blood shear stress, resulting in the release of the drug before reaching the target site [44]. The most effective strategy to increase the stability of the micelles’ formulation is to increase the hydrophobicity of the micellar core, which, in turn, decreases the CMC value. Other strategies include introducing covalent cross linking in the micellar core, stereocomplexation, and using polymers possessing ester or amide bonds [43]. Another strategy to consider is the synthesis of PCL-PEG copolymers via sequential nucleophilic substitution reactions. This method produced copolymers that self-assembled and stayed as stable polymeric micelles at pH 7.4 and showed good encapsulation ability of both hydrophobic and hydrophilic drugs [45,46]. Therefore, to design micelles for drug delivery, consideration should be given to the following elements: appropriate effect on blood components to ensure hemocompatibility, modulation of protein adsorption to reduce subsequent phagocytosis, biodistribution, and increased micellar stability in blood and extended circulation time [47].

Most studies in this review relied on electronic keyword searches, which are often regarded as the best way to conduct a systematic review [31]. However, researchers can use several complementary techniques to enhance their search efforts. First, citation tracking, which refers to efforts to find related publications based on papers that cite the article under evaluation, can augment the results since it may find other publications that ordinary database searches would otherwise miss due to the vocabulary limitation in search strategies or bibliographic records [48]. Second, reference searching, where the reference lists in the selected articles are examined for other related articles, can also be conducted. This strategy can lower the risk of missing related information when researchers encounter challenges finding related material [49]. Third, a snowballing approach, including forward and backward snowballing, can be helpful. These two methods are similar to the first two strategies mentioned. However, these three searching strategies can sometimes get out of control due to the increased number of articles being retrieved and the possibility of needing to manually appraise each article [50]. Researchers can also consider contacting experts if the specialist literature is vaguely specified [51].

## 6. Conclusions

In conclusion, the PCL-PEG copolymeric micelles with different formulations showed a remarkable performance as drug cargo for the hydrophobic anti-breast cancer drug. Copolymeric micelles conjugated with functionalised ligands enable the targeting of specific cancer cell lines. The PCL-PEG copolymer micelles showed a high drug loading and encapsulation efficiency. Additionally, the drug release of drug-loaded PCL-PEG copolymer micelles was a sustained release. Cell viability and anti-cancer efficacy studies showed that the drug-loaded copolymeric micelles have a lower toxicity and can inhibit breast cancer cell lines.

## Figures and Tables

**Table 1:** The search strings.

Database	Search String
Scopus	TITLE-ABS-KEY (“PCL-PEG” OR “polycaprolactone polyethylene glycol” OR “PCLPEG”) AND (“micelle*s” OR “micellar”) AND (“drug delivery” OR “drug cargo” OR “drug carrier”) AND (“breast cancer”)
ScienceDirect	(“PCL-PEG” OR “polycaprolactone polyethylene glycol” OR “PCL PEG”) AND (“micelle” OR “micellar”) AND (“drug delivery” OR “drug cargo” OR “drug carrier”) AND (“breast cancer”)

**Table 2:** The inclusion and exclusion criteria.

Criteria	Inclusion	Exclusion
Literature type	Journal article (empirical data)	Systematic reviews, review papers, meta-analyses, meta-syntheses, proceedings, books, book chapters, book series
Language	English	Non-English
Timeline	2016–2021	<2016

**Table 3:** The themes and sub-themes.

Authors	Synthesis and Characterisation of PCL-PEG	Preparation of Micelles	Characterisation of Micelles				Drug Release Study	Anti-Tumour Activity	
			Morphology	Particle Size Distribution and Zeta Potential	Drug Loading and Encapsulation Efficiency	DSC Analysis		Cell Viability and Cytotoxicity Study	Anti-Tumour Efficacy
Kheiri Manjili et al., (2016) [35]	/	/	/	/	/	/	/	/	/
Kheiri Manjili et al., (2017a) [36]	/	/	/	/	/	x	/	/	/
Kheiri Manjil et al., (2017b) [37]	/	/	/	/	/	/	/	/	/
Mahdaviani et al., (2017) [38]	/	/	/	/	/	x	x	/	/
Hu et al., (2017) [39]	/	/	/	/	/	/	x	x	/
Zamani et al., (2018) [40]	/	/	/	/	/	/	/	/	/
Kheiri Manjili et al., (2018) [41]	/	/	/	/	/	/	/	/	/
Peng et al., (2019) [42]	x	/	/	/	/	x	/	/	/

**Table 4:** Summary of data obtained from the synthesis and characterisation of PCL-PEG copolymers.

Authors	Amount of ε-CL	Amount of PEG	Catalyst	Reaction Condition	Drying Condition	Products Synthesised	GPC Analysis			DSC Analysis
							Mn (Da)	Mw (Da)	PDI	Melting Temperature (°C)
Kheiri Manjili et al., (2016) [35]	0.5, 1, 2, 4, 5, and 6 g	1 g	Sn(Oct)_2_, 0.01 mol	120 °C, 12 h, oil bath	23 °C, 24 h	mPEG-PCL di-block copolymer	9342–20,543	10,231–21,932	1.04–1.09	55.00
Kheiri Manjili et al., (2017a) [36]	1, 2, 4, 8, and 10 g	2 g	Sn(Oct)_2_, 0.01 mol	120 °C, 12 h, oil bath	23 °C, 24 h	PCL-PEG-PCL tri-block copolymer	8940–18,976	9731–21,321	1.05–1.12	54.72
Kheiri Manjil et al., (2017b) [37]	0.5, 1, and 2 g	1 g	Sn(Oct)_2_, 0.01 mol	120 °C, 12 h, oil bath	23 °C, 24 h	PCL-PEG-PCL tri-block copolymer	8722–11,257	10,200–15,961	1.16–1.41	52.34
Kheiri Manjili et al., (2018) [41]	2 g	1 g	Sn(Oct)_2_, 0.01 mol	120 °C, 12 h, oil bath	23 °C, 24 h	PCL-PEG-PCL tri-block copolymer	16,987	18,765	1.10	62.84
Mahdaviani et al., (2017) [38]	Not mentioned	Not mentioned	1.2 mmol EDC and 2.5 mmol NHS	Room temperature, 24 h	At reduced pressure	PCL-PEG di-block copolymer	Not stated	45,000	Not stated	-
Hu et al., (2017) [39]	Not mentioned	Not mentioned	Sn(Oct)_2_	120 °C, 48 h, oil bath	Vacuum-dried	PCL-PEG-PCL tri-block copolymer	12,875–27,844	13,615–33,084	1.057–1.218	Not stated
Zamani et al., (2018) [40]	3 g	1 g	Sn(Oct)_2_, 0.01 mol	120 °C, 12 h	Room temperature, vacuum-dried	mPEG-PCL di-block copolymer	-	-	-	57.0

**Table 5:** Summary of the preparation and characterisation of micelles.

Authour	Method Preparation	Type of Drug	Solvent Used	Selected Micelles in the Study	Size (nm)	DL %	EE %	Zeta Potential	Melting Temperature (°C)	
									Drug	Micelles
Kheiri Manjili et al., (2016) [35]	Nanoprecipitation	Curcumin (CUR)	Acetone	0.25 (CUR/copolymer mass ratio)					173.56	45.92
Kheiri Manjili et al., (2017a) [36]	Nanoprecipitation	Sulforaphane (SF)	Acetone	0.25 (SF/copolymer mass ratio)	81.70	20.65	89.32	−11.5	-	-
Kheiri Manjil et al., (2017b) [37]	Nanoprecipitation	Artemisinin (ART)	Acetone	0.25 (ART/copolymer mass ratio)	114.00	19.33	87.1	−7.57	154.35	52.34
Kheiri Manjili et al., (2018) [41]	Nanoprecipitation	Atorvastatin and rosuvastatin	Acetone	Atorvastatin-loaded	83.22	18.62	89.23	−15.45	167.73	55.48
Mahdaviani et al., (2017) [38]	Cosolvent evaporation	Cabazitaxel (CBZ)	Acetone	Rosuvastatin-loaded	55.66	20.0	88.19	−7.72	75.51	51.12
Hu et al., (2017) [39]	Thin-film hydration and ultrasonic dispersion	Paclitaxel (PTX)	Methylene chloride	CBZ-loaded	53.72	13.21	59.01	−2.99	-	-
Zamani et al., (2018) [40]	Cosolvent evaporation	Tamoxifen (TMX)	Acetone	PTX-loaded	110.00	8.5	82.5	Not mentioned	-	-
Peng et al., (2019) [42]	Emulsion solvent evaporation	Paclitaxel	Chloroform	1:6 (TMX/copolymer mass ratio)	255.80	8.87	87.97	−17.9	142.00	55.00

**Table 6:** HPLC parameters for drug loading and encapsulation efficiency analysis.

Authors	Special Solvent	Mobile Phase	Column	Temperature	Flow Rate	Sample Injection Volume	Sample Detection
Kheiri Manjili et al., (2017a) [36]	-	Acetonitrile and water (45:55, *v*/*v*)	C18 analytical column (250 mm × 4.6 mm, particle size 5 µm)	Not mentioned	1.0 mL/min	20 µL	λ max = 241 nm, SF
Kheiri Manjil et al., (2017b) [37]	-	Methanol and 5% (*w*/*v*) acetic acid (70:30, *v*/*v*)	C18 analytical column (150 mm × 4.6 mm, particle size 5 µm)	Not mentioned	1.0 mL/min	20 µL	λ max = 420 nm, ART
Mahdaviani et al., (2017) [38]	Acetonitrile (to dissolve CBZ)	Methanol	Agilent ZORBAX Eclipse Plus C18 column (5 μm, 4.6 mm × 150 mm)	Not mentioned	1.0 mL/min	Not mentioned	λ max = 248 nm, CBZ
Hu et al., (2017) [39]	Acetonitrile (to dissolve PTX)	Acetonitirle and water (50:50, *v*/*v*)	Reverse-phase column (Symmetry, 150 mm × 4.6 mm, five μm)	40 °C	1.0 mL/min	Not mentioned	λ max = 227 nm, PTX
Peng et al., (2019) [42]	Acetonitrile (to dissolve PTX)	Acetonitrile and water (50:50, *v*/*v*)	C18 column (5 μm, 4.6 × 150 mm)	30 °C	1.0 mL/min	Not mentioned	λ max = 227 nm, PTX

**Table 7:** Drug release study of drug-loaded PCL-PEG copolymers.

Authors	Release Medium	pH	Molecular Weight Cut-Off	Incubation Temperature	Shaking Speed	Method of Drug Concentration Analysis	Cumulative Drug Release
Kheiri Manjili et al., (2016) [35]	PBS with 5% (*v*/*v*) Tween 80	7.4	120,000 Da	37 °C	Not mentioned	UV-Vis	~45.32%
		5.5					~76.8%
		Human plasma					~63.21%
Kheiri Manjili et al., (2017a) [36]	PBS	7.4	120,000 Da	37 °C	Not mentioned	HPLC	~56.75%
		5.5					~65.75%
		Human plasma					~63.21%
Kheiri Manjil et al., (2017b) [37]	PBS with 2% (*v*/*v*) tween 80	7.4	120,000 Da	37 °C	Not mentioned	HPLC	~38.0%
		5.5					~50.0%
		Human plasma					~42.0%
Kheiri Manjili et al., (2018) [41]	PBS with 5% (*v*/*v*) Tween 80	7.4	12,000 Da	37 °C	Not mentioned	UV-Vis	~55.76 %
		5.5					~60.12 %
Zamani et al., (2018) [40]	PBS	7.4	140,000 Da	37 °C	100 rpm	UV-Vis	~25.0%
		5.5					~55.0%
Peng et al., (2019) [42]	PBS with 1% Tween 80	7.4	10,000 Da	37 °C	120 rpm	HPLC	~60.0%
		6.5					~63.5%

**Table 8:** Pharmacokinetic study of drug-loaded PCL-PEG copolymeric micelles.

Authors	Solution	Dose	Values of Area under the Plasma Concentration—Time Curve (AUC0-∞)	Apparent Volume of Distribution (Vd)
Kheiri Manjili et al., (2016) [35]	CUR aqueous solution	50 mg/kg	8.734 ± 1.09 h ng/mL	8769.132 ± 1321.3 L/kg
	CUR-loaded micelles		488.17 ± 1.23 h ng/mL	807.123 ± 342.9 L/kg
Kheiri Manjili et al., (2017a) [36]	SF aqueous solution	30 mg/kg	8.3425 ± 1.564 h ng/mL	9420.132 ± 2221.3 L/kg
	SF-loaded micelles		465.87 ± 34.2 h ng/mL	909.123 ± 252.1 L/kg
Kheiri Manjil et al., (2017b) [37]	ART aqueous solution	25 mg/kg	320 ± 4.02 h ng/mL	-
	ART-loaded micelles		5234 ± 1.13 h ng/mL	-
Peng et al., (2019) [42]	PTX-PCL-PEG-Her	5 mg/kg	1922.78 μg * h/L	6.59 L/kg
	PTX-PCL-PEG		1874.83 μg * h/L	11.93 L/kg
	TAXOL^®^		5591.67 μg * h/L	3.32 L/kg
